# Class imbalance in multi-resident activity recognition: an evaluative study on explainability of deep learning approaches

**DOI:** 10.1007/s10209-024-01123-0

**Published:** 2024-06-13

**Authors:** Deepika Singh, Erinc Merdivan, Johannes Kropf, Andreas Holzinger

**Affiliations:** 1 Institute for Medical Informatics, Statistics and Documentation, Medical University Graz, Graz, Austria; 2 AIT Austrian Institute of Technology (AIT), Vienna, Austria; 3 Human-Centered AI Lab, Institute of Forest Engineering, Department of Forest and Soil Sciences, University of Natural Resources and Life Sciences Vienna, Vienna, Austria

**Keywords:** Human activity recognition, Multiple residents, Class imbalance, LSTM, BiLSTM networks, Trust, Explainability

## Abstract

Recognizing multiple residents’ activities is a pivotal domain within active and assisted living technologies, where the diversity of actions in a multi-occupant home poses a challenge due to their uneven distribution. Frequent activities contrast with those occurring sporadically, necessitating adept handling of class imbalance to ensure the integrity of activity recognition systems based on raw sensor data. While deep learning has proven its merit in identifying activities for solitary residents within balanced datasets, its application to multi-resident scenarios requires careful consideration. This study provides a comprehensive survey on the issue of class imbalance and explores the efficacy of Long Short-Term Memory and Bidirectional Long Short-Term Memory networks in discerning activities of multiple residents, considering both individual and aggregate labeling of actions. Through rigorous experimentation with data-level and algorithmic strategies to address class imbalances, this research scrutinizes the explicability of deep learning models, enhancing their transparency and reliability. Performance metrics are drawn from a series of evaluations on three distinct, highly imbalanced smart home datasets, offering insights into the models’ behavior and contributing to the advancement of trustworthy multi-resident activity recognition systems.

## Introduction

Human activity recognition in an intelligent environment is a highly dynamic research area which has gained a lot of attention due to its varied applications. The applications of activity recognition systems are categorized as: active and assisted living systems for smart homes (SH), monitoring and surveillance systems for indoor and outdoor, health care monitoring and tele-immersion applications [1–3]. Among these, SH plays an important role, especially in user behavior analyses, health monitoring, and assistance. Most of the research on activity recognition in SH has investigated single resident activity monitoring [4–7]. However, in real-life scenarios, a home is not always occupied by a single resident but often occupied by more than one resident. Therefore, developing an SH solution from the perspective of multiple residents is extremely crucial.

In recent years, there has been an increase in multiple occupancy-based research related to activity modelling and data association. However, there are still various challenges to be addressed in multiple occupancies, such as finding the suitable models for data association i.e. identification of the residents by whom each sensor is triggered and capturing interactions between the occupants [8]. Another major challenge that occurs while developing real-life applications is the class imbalance problem. Activity recognition is mainly considered a classification problem where the performance of the system depends on the model selection, features involved, number of classes, and the size of the datasets available for training the system. In most of the SH datasets, there is a lack of uniformity in different daily living activities of residents; which is obvious as in real-life situations, some activities are performed more often than others.

Although several studies have been conducted for class imbalance, there remains a lack of empirical work on addressing the class imbalance in multiple residents activity recognition. In this work, we report an empirical study of both data-driven and algorithm-driven techniques for handling class imbalance. Data-driven approaches modify the original dataset by oversampling the minority samples and can provide a balanced distribution without losing information on the majority class. Undersampling techniques alter the dataset by removing samples from the majority class. The main advantage of undersampling lies in the reduction of the training time, which is significant in the case of highly imbalanced large datasets [9]. In algorithm level techniques, we employed cost-sensitive learning to deep learning models, which has performed well as reported in previous works in class imbalance problem. However, the majority of works use statistical methods such as SVM and Naive Bayes as a base classifier in cost sensitive learning approach [10]. In other works, machine learning methods have been used in activity recognition which relies on feature extraction techniques including time-frequency transformation and statistical approaches. In such methods, the extracted features are carefully engineered and heuristic. There is no universal feature extraction method that can effectively capture distinguishable features of human activities. Consequently, we selected the Long Short-Term memory (LSTM) network as it allows extracting highly discriminative non-linear feature representations while modeling temporal sequences by learning long-term dependency. In addition, LSTM and 1D-convolutional neural network outperformed other statistical machine learning models on single resident activity recognition [11].

To summarize, the main contributions of this paper are:

i. a review on handling class imbalance problem with deep learning and explainable AI (XAI);
ii. employing LSTM and BiLSTM networks for multiple resident activity recognition;
iii. evaluating model performance by taking each resident separately and also with combined activity labels of the residents;
iv. conducting extensive experiments using both data level and algorithm level class imbalance techniques; and,
v. investigating model performance at different sample ratios and cost coefficients on three benchmark datasets.

The paper is further structured as follows: Sect. 2 reports the related works. Section 3 introduces the SH datasets, LSTM and BiLSTM network and different data imbalance methods that are used in the paper and Sect. 4 describes experiments performed. The results of the paper are demonstrated and discussed in the next section, which is followed by a concluding section highlighting the major findings.

## Related work

In this section, we review related works on multiple resident activity recognition and imbalanced data classification approaches and discuss them in detail which eventually laid the foundation of the current work.

### Multiple resident activity recognition

Activity recognition has been categorized mainly into two approaches: Vision based [12–14] and pervasive sensing based [15–17]. Vision-based activity recognition can provide good results but have raised various privacy concerns among the residents due to required camera installations in their private spaces [18, 19] whereas pervasive sensing-based activity recognition approaches use data from wearable sensors and non-intrusive environment sensors [20]. A significant amount of work has been performed on activity recognition using wearable sensors. A new technology called Body Sensor Networks (BSN) has emerged which consists of different wearable sensors that capture and process physiological signals on the human body. BSNs then collect data from wearable sensors and process them to extract useful information [21, 22]. A major issue with wearable sensors is that wearing or carrying a tag is often not feasible especially with the old people, who often forget to wear, or not willing to wear tags at all. There have been efforts to create adaptive solutions for user adoption and integration. Nonetheless, the challenge of usability persists among older individuals [23–25]. Pervasive sensing using environment sensors offers the advantage of being non-intrusive to the inhabitants and do not require to carry any tag or device. In pervasive sensing, the sensors are deployed in the environment and capture activities of the residents, which then can be used for activity recognition. But there are some challenges in this approach as well. Recognizing human activities using environment sensors is challenging because sometimes the data captured by the sensors can be disturbed from the surroundings which can make data noisy and human activities are complex. In such a case, sensor deployment, its configuration, and selection of the classification model play an important role in the identification of activities of residents and the residents themselves [26].

In previous works, diverse computational models have been applied in the context of single resident activity recognition which includes standard data mining approaches, probabilistic models, and machine learning models such as neural networks, support vector machines, decision trees, and ontologies. However, for multi-resident activity recognition, such a diversity of models has not been used yet. The problem in multiple resident activity recognition using non-intrusive sensors is the association of sensor data when such sensors cannot directly identify residents and interactions between them, whereas, in a single resident setting, sensors’ states reflect directly the activity of the sole resident. Multiple residents’ activities can have different scenarios: the same activity can be performed by two or more residents (e.g. eating a meal or watching TV together) or multiple residents perform different activities independently (e.g. one resident is watching TV and the other preparing meal). Evidently, there is a need for a model that is capable of capturing the complex nature of both joint and independent activities. Previous works have addressed multiple resident activity recognition using wearable sensors such as RFID [17], accelerometer [27] and videos [28]. Machine learning approaches used previously for multi-resident activity recognition are naive Bayes, Markov Model classifier [29] and conditional random field (CRF) [30] on CASAS [31] dataset in which data association problem was investigated. In [32], the authors proposed a two-stage activity recognition method in order to exploit more knowledge in multi-resident activities. The two phases in the model include the building phase and activity recognition phase and it converts multi-label problems into a single-label problem by treating activities of residents as combined label state using HMM (Hidden Markov Model) and CRF (Conditional Random Field) classifiers. In recent works, deep learning models have shown impressive performances in various fields. LSTM network which is variants of recurrent neural network (RNN) is good at solving time series problems as its design enables gradients to flow through time readily [33]. Deep Residual Bidirectional LSTM network has been used for activity recognition using wearable sensors on UCI dataset (which uses data from a smartphone) and Opportunity dataset (data from wearable, object, and ambient sensor) [34]. In [35], CNN (Convolutional Neural Network) and LSTM have been used for extracting Spatio-temporal features from multisensory and multimodal data which includes RFID, audio data, and videos for concurrent activity recognition. In [36], a joint diverse temporal learning framework using LSTM and 1D-CNN models has been proposed to improve human activity recognition.

However, the existing state of the art approaches in multi-resident activity recognition focuses on the improvement of recognition algorithms using accuracy as performance metrics rather than handling imbalanced dataset. Furthermore, there is a lack of comprehensive studies on how different class imbalance approaches perform in the multiple residents’ activity recognition domain.

### Imbalanced data classification

Existing research uses various machine learning and deep learning models for activity classification but lacks in analyzing the class imbalance problems in the dataset, through which a model achieves very high accuracy but did not reveal the actual performance of the model due to class imbalance. Likewise, in some machine learning problems, not every mistake is treated equally. This is very true in the SH setting; for example, if the system makes a mistake in detecting a resident fall, it is much more harmful than making a mistake in detecting if a user is brushing his teeth. Training with equal importance for each activity in the home environment is not suitable to provide high user experience and satisfaction. In the multi-resident setting, common methods to help with the imbalance dataset needs to be altered since instead of one classification there are multiple classifications (for each resident of the house).

Three major approaches have been defined to learn imbalanced data [37]: data-level methods, algorithm level methods, and hybrid methods. Data level methods concentrate on modifying training sets to balance the data distributions by adding or removing samples. Such methods use oversampling (addition of new sampling to minority class) and undersampling (removing samples from majority class) approaches for balancing the data distributions. This way, data level approaches are used to avoid the modification of the learning algorithm by decreasing the effect caused by an imbalance with a preprocessing step [38]. Synthetic Minority Over-sample Technique (SMOTE) is the popular oversampling method [39] with an idea to create new minority samples by interpolating several minority class instances that lie together. The strategy used in SMOTE is problematic as it blindly generalizes the minority class without regard to the majority class, particularly in the case of highly skewed class distribution where the minority class is very sparse in comparison to the majority class, thus resulting in a high chance of class mixture [40]. In undersampling techniques, four K-Nearest Neighbour (KNN) methods [41], namely, NearMiss-1, NearMiss-2, NearMiss-3, and the “most distant” method were proposed, in which instead of using the entire set of majority samples, a small subset of these samples was selected such that resulting training data is less skewed. Results of the experiment suggest that the NearMiss-2 method provides competitive results in comparison to SMOTE and random undersampling. Algorithm level methods modify existing learning algorithms to alleviate the bias towards majority classes. These methods require special knowledge of both the learning algorithm and the application domain, comprehending the reason behind the failure of the classifier when the class distribution is uneven. The most popular of such methods are cost-sensitive approaches [42]. In such approaches, the given learning algorithm is modified by incorporating varying costs for each of the considered groups of samples. Another algorithm level approach is one-class learning which focuses only on the target group and thus helps in eliminating the bias towards any group. Hybrid methods combine data-level methods and algorithm level methods by extracting strong features from both the approaches, merging data level solutions with classifier ensembles is one of the widely used hybrid approaches [43]. There exist some works that propose hybridization of cost-sensitive learning and sampling methods [44].

Numerous other works have been performed for handling class imbalance in traditional classification problems using data preprocessing and algorithm level techniques [45–47]. These studies have shown that for the various base classifiers, a balanced dataset provides improved overall performance compared to an imbalanced dataset. Traditional machine learning algorithms such as SVM, Decision tree, Naive Bayes, Random forest, Hidden Markov Models, and their ensembles were used to balance between minimizing the total recognition error and maximizing the accuracy of classification on minority class [48]. The major drawback of these methods is that they rely on handcrafted and classical heuristic feature extraction techniques. Recently, deep learning methods have shown promising results in various applications such as natural language processing, image classification, speech recognition, and also in human activity recognition systems by outperforming on raw sensor datasets [4].

#### Handling class imbalance in deep learning

Some of the works with deep learning methods for handling class imbalance use CNN for representation learning [49] and proposed quintuplet sampling with a triple-header loss for imbalanced learning. Another work proposed Deep Over-sampling (DOS) with CNN architecture [50] to address the effect of class imbalance on both classifier and representation learning. Empirical results of the proposed DOS framework showed improvement in addressing the class imbalance problem. A new loss function in a deep neural network is proposed in [51] which captures classification errors from both majority and minority classes. Another method was presented in [52] to optimize the network parameters and class sensitive costs. Deep reinforcement learning has shown promising results in various applications, therefore recent work also explores the performance of deep reinforcement learning model for imbalanced classification and evaluated their approach on text and image data [53] where classification problem was formulated as a sequential decision-making process. The environment returns a high reward to minority class samples but a low reward to the majority class sample and the process was terminated when the agent misclassifies the minority class sample. Deep Q-learning was used to find the optimal classification policy for the Imbalanced Classification Markov Decision Process (ICMDP). Experiments showed better classification performance on imbalanced text datasets. The survey on class imbalance for deep learning presents classical methods such as random oversampling and cost-sensitive target function, which show promising results when applied in deep learning situations [54]. In general, reinforcement learning (RL) is highly relevant because it is very close to a human-in-the-loop approach and can therefore be useful to bring human conceptual understanding into the machine learning pipeline [55].

#### Class imbalance problem with XAI and interpretable machine learning (IML)

Interpretable and explainable ML models are providing promising solutions in various critical fields such as healthcare, finance, and computer vision as compared to traditional methods. Explainable AI focuses on the explanation of learning models and machine learning interpretability allows users to perceive the results of learning models by giving the reasons for the predictions that it has arrived at. State-of-the-art models like Deep Learning and Boosting classifiers are trained to classify instances with high accuracy, while their interpretability is enhanced through eXplainable AI (XAI) techniques such as Layerwise Relevance Propagation (LRP) [56], SHAP [57], and LIME [58]. For an overview and comparison of XAI methods see: [59]. XAI has meanwhile developed into an established, large and complicated field with many different approaches. Due to the many different XAI techniques, it is not so easy for the non-expert data scientist to decide which method is best to use. This is where design patterns can help [60]. The LRP process mentioned above is an example of such a design pattern: Decomposition: Breaking down complex elements into smaller, more understandable parts and explaining how they work together to reach a final decision or result. Explainability is essential for fostering trust, facilitating human understanding, and ensuring ethical and effective decision-making in high-stakes applications [61]—finally to ensure that the human remains in control of AI [62].

Performance evaluations, including confusion matrices, affirm the models’ efficacy and reliability in classifying high-cost, minority-class instances. Interpretation is a critical task in both imbalanced data learning and IML; however, both techniques have different perspectives. When applied in deep learning, imbalanced learning has typically sought to understand the class imbalance with overlap, sub-concepts and data outliers. Whereas, IML methods are designed to explain internal representations, inputs and outputs in the neural networks. In addition, imbalanced learning is generally concerned globally for all the classes; whereas IML learning explain model decisions with respect to specific instances [63]. Therefore, it would be interesting to combine both fields into a single framework to better understand the predictions made by a model concerning imbalanced data.

There is few research on the potential effect of class imbalance on model-agnostic interpretation methods. Patil et al. (2020) [64] address the challenge of imbalanced datasets in AI by employing Synthetic Minority Oversampling Technique (SMOTE) for data resampling and applied LIME and SHAP to balanced dataset to identify the important features. They determined that oversampling does not change the correlation among features, as the crucial features for predicting both valid and fraudulent observations remain consistent. Several research studies have assessed the reliability of LIME and SHAP [65, 66], yet none of them have considered the issue of class imbalance. The impression could be that the class imbalance might not affect the consistency of interpretations or could even reduce uncertainty, as interpretations may come from the majority class with more stable distributions. Conversely, it could potentially hinder interpretation methods’ performance, as rare events might fall outside the typical distribution, making them challenging to interpret. Chen et al.(2024) [67] argue that the class imbalance does have an adverse effect on the interpretive performance of both LIME and SHAP. The findings indicate that interpretations generated from LIME and SHAP are less stable as the class imbalance increases, suggesting that class imbalance negatively impacts the interpretability of machine learning models. The potential effects of imbalanced learning techniques on the performance of interpretation methods need to be investigated. Resampling methods can tackle imbalanced data challenges, however, they cause problems of overfitting (over-sampling methods) or information loss (under-sampling methods). Exploring the cost-sensitive learning relationship with the interpretability of a network model involves understanding how these cost adjustments influence model decisions and interpreting these decisions, particularly through the lens of explainable methods. SHAP can help in understanding the impact of class weights and misclassification analysis. Based on the insights from the SHAP analysis, the model can be refined by adjusting the class weights if certain features are disproportionately influencing the model, leading to biases and re-evaluating the balance between managing misclassification costs and maintaining model interpretability. Furthermore, it would be beneficial to theoretically examine how class imbalance affects the stability of interpretation methods. For instance, we could initiate our investigation by employing Logistic Regression as the predictive model and establishing theoretical outcomes regarding interpretations generated by SHAP or LIME in the presence of class imbalance. Such an approach could provide further insights into evaluating interpretation method stability, especially when dealing with complex "black-box" machine learning models. However, conducting an experimental study on the integration of interpretable models and its effect on class imbalance falls beyond the scope of this paper.

#### Handling class imbalance in activity recognition

Few studies have been performed in single resident activity recognition using improved SMOTE algorithm to address issues concerning imbalanced activity classes [68]. SMOTE [39] is the widely used algorithm as it creates new non-replicated examples by interpolating neighboring minority class instances but it fails to preserve the class covariance structure and increases overlapping between classes. Another work uses a cost-sensitive SVM approach for the classification of activities and compared the results with HMM, CRF, and traditional SVM models [69]. Most of the works on handling imbalance classes focus on vision and text classification problems but very less work has been performed in handling class imbalance in multiple resident activity recognition. In addition, existing works lack comparative studies of different class imbalance approaches.

Therefore, this paper presents a comprehensive study of both data level and algorithm level class imbalance approaches in multiple resident activity recognition. Since temporal deep learning methods have shown promising results on raw sensor datasets in single resident activity recognition, we used LSTM and BiLSTM networks as a classifier for addressing the class imbalance in activity recognition.

## Methodology

### Smart home datasets

In this work we have used publicly available ARAS [70] and CASAS-Kyoto Multiresident ADL Activities datasets (fourth number dataset on CASAS dataset list: http://casas.wsu.edu/datasets/) [16, 71]. ARAS is a widely used dataset in activity recognition systems whereas the CASAS-Kyoto Multiresident ADL Activities dataset has not been used much in previous works. As the collection of real SH data is time-consuming, costly, and difficult to annotate, the publicly available datasets are used to provide a baseline for comparison.

#### ARAS multi-resident ADL dataset

**Fig. 1:**
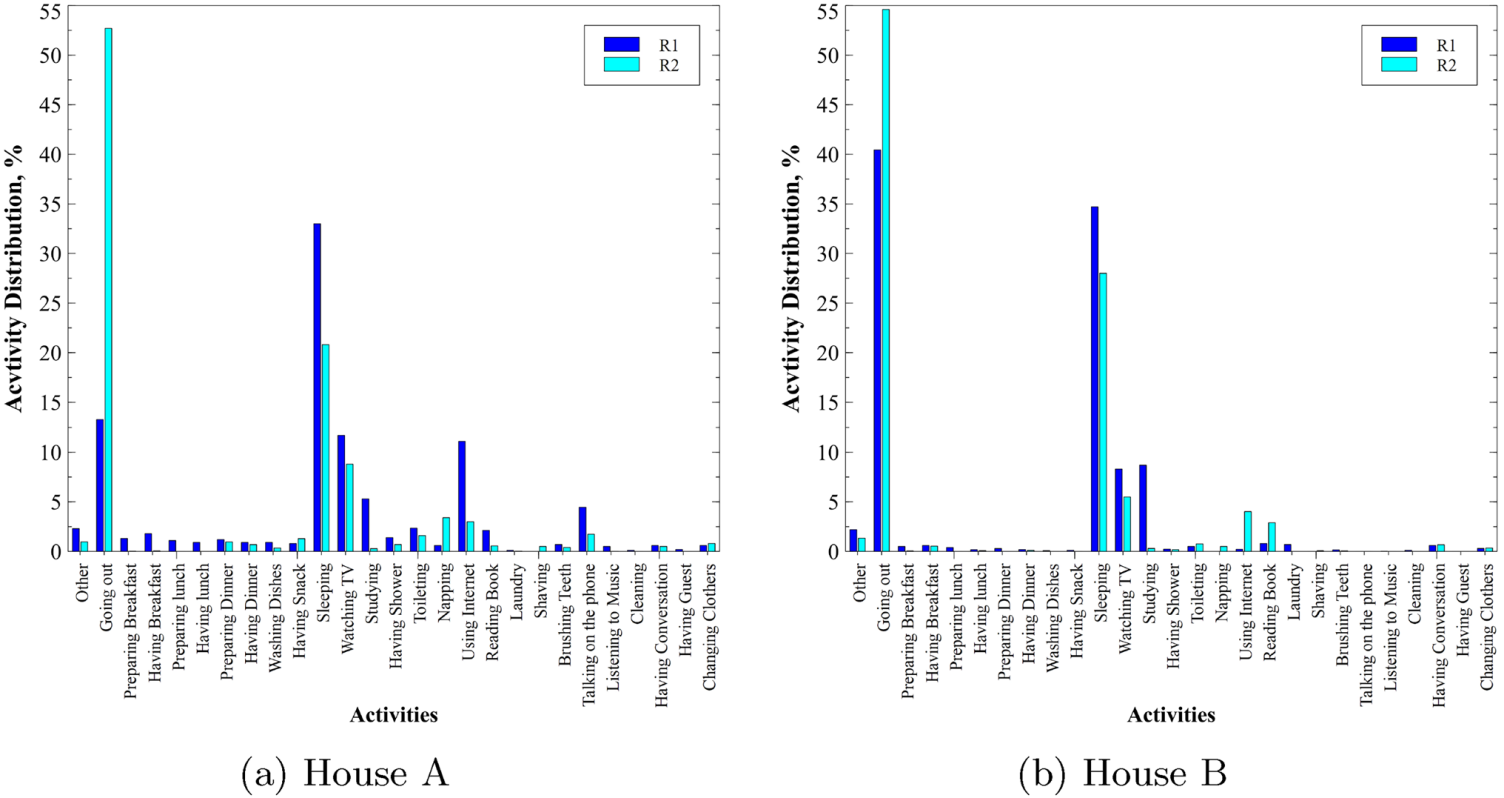
Activity distribution of both residents (R1 and R2) in the ARAS dataset

ARAS datasets use ambient sensors such as contact sensor, temperature sensor, sonar distance sensor, force sensor, photocells, resistors, and infrared receivers in the SH setting. The dataset consists of 20 different types of sensor signals as features together with the activity labels of two residents for two different houses which are termed as House A and House B. Each house has 30 days of a dataset with 30 separate files for a month and every file contains 86,400 instances. The dataset consists of 27 different types of activities for each resident. The distribution of activities in House A and House B of the ARAS dataset are shown in Fig. 1.

As visible from Fig. 1 the dataset of both the residents in two houses is highly imbalanced where only few activities in the distribution are more than 35% and most of the activities are less than 10% of the whole dataset.

#### CASAS-Kyoto multiresident ADL activities dataset

**Fig. 2:**
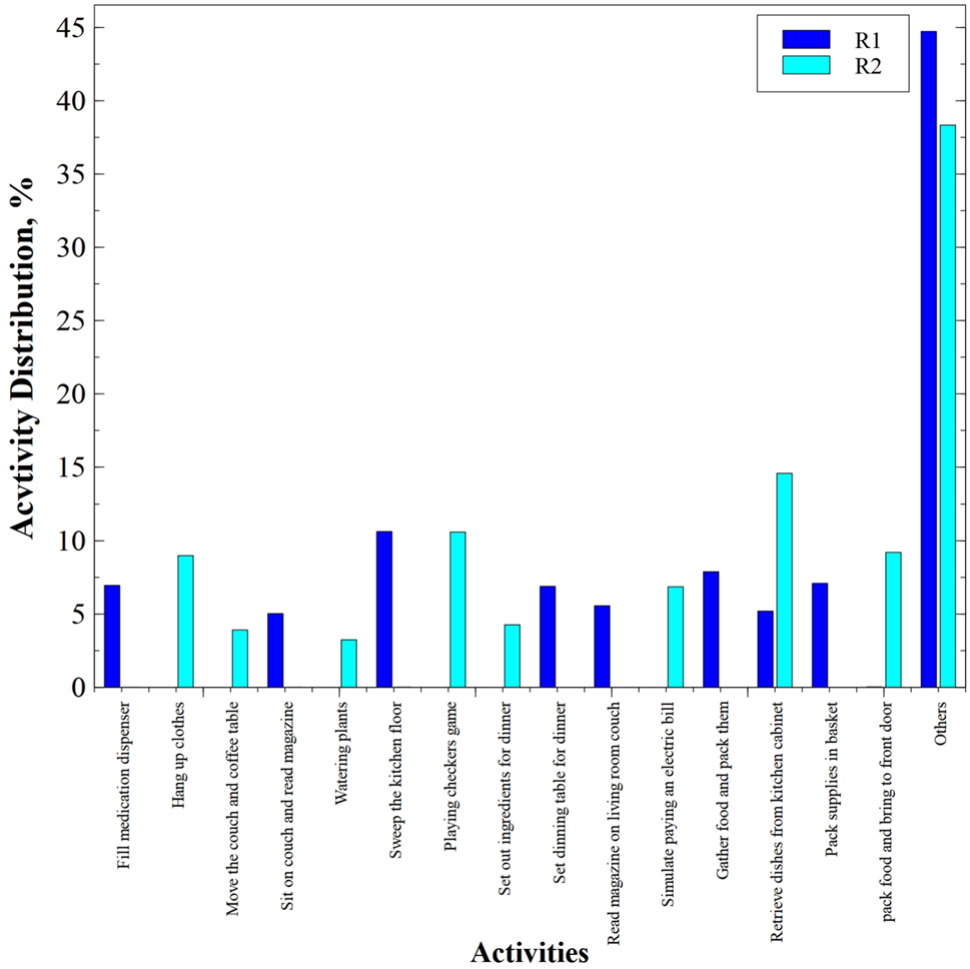
Frequency count of activities in the CASAS-Kyoto Multiresident ADL Activities dataset

The CASAS-Kyoto Multiresident ADL Activities dataset was collected in a smart apartment testbed located at Washington State University (WSU). The sensors used in the dataset are motion, item, cabinet, water sensors, burner, phone, and temperature sensors. The smart space was occupied by two residents at the same time where they performed daily living tasks concurrently. The collected sensor events were labeled with activity and person identifications. The dataset has 15 different daily living activities that were performed by both residents, in which few activities (moving furniture, playing checkers, paying bills, and packing picnic supplies) were jointly accomplished by both residents. Since some activities were performed jointly by both the residents and some individually, when an activity is performed by only one resident, there is no label for the activity of the other resident. As both residents are present in the apartment, we assigned a label (named as "Other") to the activity of the second resident which is not known, which makes our dataset of 16 activity labels for both residents. However, in many cases, there were sensor readings for both residents and their activity labels. The frequency distribution of activities in the dataset is shown in Fig. 2.

### LSTM models

LSTM networks [72] are a successful extension of RNNs designed to avoid the long-term dependency problem associated with RNN. LSTM models introduce a new state, called cell state and constant error carousel (CEC) that allows constant propagation of error signals over time, thus solving the problem of vanishing gradients. In addition, LSTM uses a gating mechanism over an internal memory cell to control access to CEC and to learn a more complex representation of the long-term dependencies. LSTM is better at classifying, processing, and predicting time series data with the time lags of unknown sizes. An LSTM block consists of input, output, and forget gates which are responsible for write, read and reset operations respectively for the memory cell. The main component of LSTM is the memory cell which is responsible for remembering states for short or long periods over arbitrary time intervals. Each LSTM cell operates as a memory to write, read, and erase information based on the outcomes rendered by input, output, and forget gates respectively. Forget gate receives new time step $X_{t}$ and previous output $h_{t-1}$ as input and gives output using sigmoid activation function to decide which information will be kept or deleted. The information will be deleted if the output of the sigmoid activation function is 0, while information will be kept if the output is 1. The forget gate computation is shown in Eq. (1). The next step decides what new information will be stored in the cell state. This step has two parts, first, the input gate layer decides which new information from the current input ($X_{t}, h_{t-1}$) is updated to the cell state. In the second step, tanh activation function that generates a new candidate value $\tilde{C_{t}}$, could be appended to the cell state. The multiplication of these two parts will be added to the multiplication of forget gate ($f_{t}$) with the previous cell state ($C_{t-1}$) to generate a new cell state ($C_{t}$). The forget gate ($f_{t}$) is multiplied with the previous cell state ($C_{t-1}$), forgetting the information which was specified to be deleted earlier. Then we append $i_{t} * \tilde{C_{t}}$, which is the new candidate value, scaled by how much the cell state is updated. The computation of the input gate, new candidate value and cell state is shown in Eqs. (2)–(4). In the final step, the output gate is computed based on the filtered version. First, the previous hidden state and the current input time step are passed to the sigmoid activation function, and then the new state is put through $\tanh$ function. Then the output of the sigmoid function is multiplied with the output of $\tanh$ function to generate the next hidden state. The update cell state and new hidden state forward the information to the next time step. Equations (5) and (6) shows the computation of output gate and hidden state ($h_{t}$).

1 $$\begin{aligned} f_{t}&= \sigma (W_{f}\cdot [h_{t-1}, x_{t}] + b_{f}) \end{aligned}$$

2 $$\begin{aligned} i_{t}&= \sigma (W_{i}\cdot [h_{t-1}, x_{t}] + b_{i}) \end{aligned}$$

3 $$\begin{aligned} \tilde{C_{t}}&= \tanh (W_{C}\cdot [h_{t-1}, x_{t}] + b_{c}) \end{aligned}$$

4 $$\begin{aligned} C_{t}&= f_{t} * C_{t-1} + i_{t} * \tilde{C_{t}} \end{aligned}$$

5 $$\begin{aligned} o_{t}&= \sigma (W_{o}\cdot [h_{t-1}, x_{t}] + b_{o}) \end{aligned}$$

6 $$\begin{aligned} h_{t}&= o_{t} * \tanh (C_{t}) \end{aligned}$$

where $\sigma$ is the sigmoid activation function, $\tanh$ is hyperbolic tangent function, x is the input data and W is the weight matrix. The LSTM equations are adapted from [73].

**Fig. 3:**
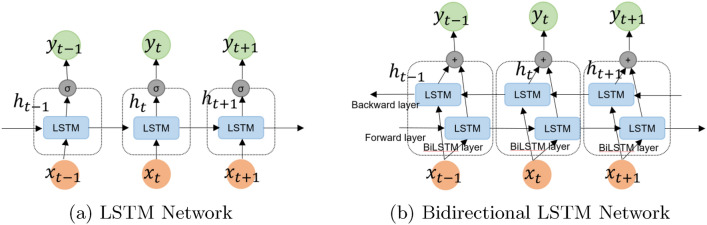
LSTM and Bidirectional LSTM

The architecture of LSTM and BiLSTM network is shown in Fig. 3. The input layer of the network comprises an embedded vector that contains a sequence of sensor events and then *n* LSTM cells are fully connected to the inputs and have recurrent connections with the other LSTM cells.

Finally, a dense output layer of the network performs the classification task. In the BiLSTM network, two parallel LSTM are used for forward and backward loops, which extracts patterns from the past and future events. The forward layer reads the input from the left to right direction whereas the backward layer reads the input from right to left direction.

The output prediction is the weighted sum of the prediction score from both the forward and backward layer. In both networks, the Adam optimizer is used for training the network and minimizing the softmax cross-entropy loss function.

### Handling class imbalance with LSTM and BiLSTM networks

In this paper, the following three methods are used with LSTM and BiLSTM networks.

#### Oversampling

Oversampling is the data level approach that aims to balance the class distribution by increasing samples of the minority class. Oversampling is performed by computing the sampling ratio (also known as the class imbalance ratio) between the minority class and majority class. We selected the most frequent activity and reduced the imbalance of less frequent activities in the training set. We oversampled less frequent activities with varying sampling ratios but we never, in any case, oversampled less frequent activity to the amount where it was more frequent than the actual most frequent activity. For example, suppose Resident 1 has 1000 samples and Resident 2 has 5000 samples and maximum activity is 10,000; in this case, we threshold oversample at 2, even though we could apply oversample by 10 (if only Resident 1 was taken into consideration). We used different sampling ratios (from range 1 to 10) and conducted experiments over these ranges. The optimal difference in model performance was observed at sampling ratios 2 and 5.

#### Undersampling

Similar to oversampling, undersampling is also a data level approach performed by computing sampling ratio where we reduced the samples of most frequent activities of the residents. We limited the undersampling ratio in a way that the most frequent activity will still be the most frequent even after being undersampled. For example, if any of the Resident 1 or Resident 2 activity ratios are lower than average (uniform distribution for all activities in the original count) we keep these instances and do not undersample. We only undersample if both activities are over-represented and again keeping in mind that we threshold undersampling ratio taking into account average. We also tried different sampling ratios from range 0.25 to 1.0 and conducted experiments over these ranges, however, the optimal difference was observed at 0.25 and 0.5 undersample ratio.

Data level approaches are not dependent on the classifier as they avoid the modification of the learning model by reducing the effect caused due to imbalanced data with a preprocessing step. Thus, these approaches are more versatile.

#### Cost-sensitive learning

Cost-sensitive learning lies between data level and algorithm level approach as it incorporates both data-level processing by adding costs to samples and algorithm level modifications by modifying the learning process [74]. This method evaluates the cost associated with the misclassifying samples. It does not create a balanced data distribution, rather assigns the training samples of different classes with different weights, where weights are in proportion to the misclassification costs. In the cost-sensitive version, we scaled the loss according to the cost coefficients in frequent activities and limit the ratio of cost coefficient below the ratio of most frequent/given activity frequency. In this approach, we also conducted experiments with different cost coefficients (from range 1 to 10), and the best model performance was observed at cost coefficients 2 and 5.

**Fig. 4:**
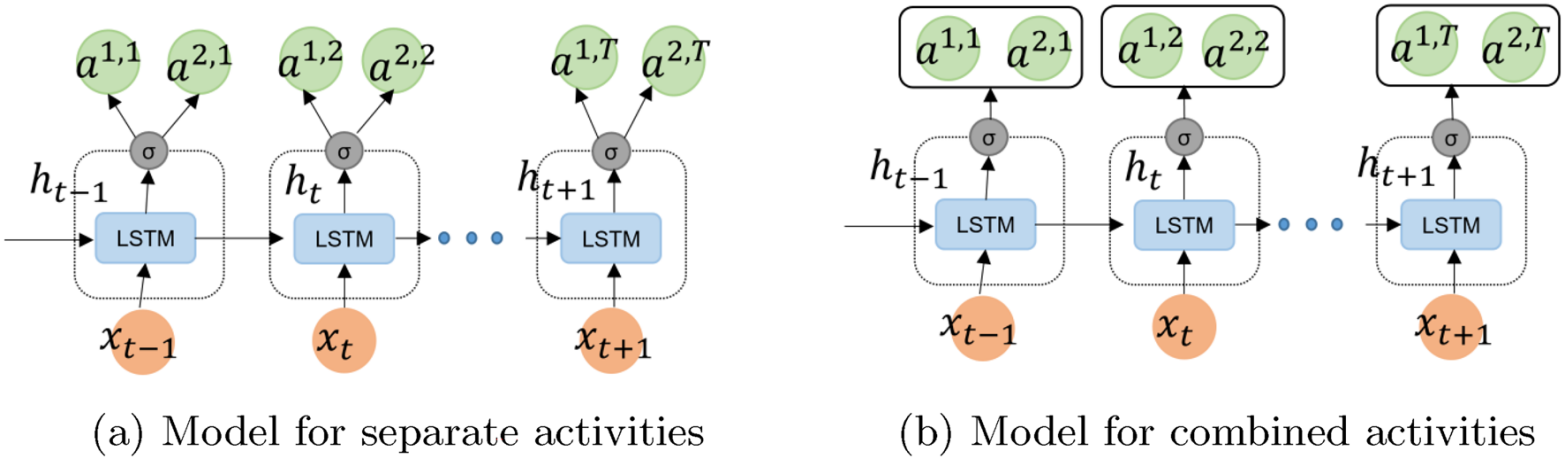
LSTM model for multi-resident activity recognition

Since the dataset contains multiple residents where most of the activities are performed separately by each resident but some activities are performed together as well, we looked at how often each resident does each activity by themselves, and we also looked at how often they do activities together. Figure 4 depicts the LSTM model for multiple residents activity recognition with activity labels $a^{1,1}a^{2,1}$,...,$a^{1,T}a^{2,T}$, where $a^{1,1}$ is activity label of first resident and $a^{2,1}$ is activity label of second resident and similarly for all the labels. Figure 4a shows the LSTM model with activity of each resident separately and Fig. 4b shows the model with combined activities of residents. For example, in the case of separate activity labels, we selected activity 1 and activity 3 separately for different residents and applied class imbalance methodologies for users separately, where we always kept the most frequent activity samples more than any other activity. In the case of combined activities of both residents, we used a tuple of activities and calculate the frequency of tuple activities, such as activity (1, 3), and applied class imbalance methodologies to these tuple activities.

Oversampling the activities with sampling ratio = 2 or 5, does not represent multiplying each resident activity with sample ratio 2 or 5. We took into consideration that increasing one resident activity will also change the distribution of other resident activity as in the dataset we have sensor information for both the residents together. Similarly, while undersampling the dataset with a sampling ratio = 0.25 and 0.5 does not mean reducing the activity distribution to one-fourth and half. Instead, we performed sampling such that when we undersample with 0.25 sampling ratio, we selected 0.25 probability if a certain data point should be added or not. Therefore, the exact distribution of activities may vary in each case.

## Experiments

The experiments were performed on three SH datasets, in which two houses (House A and House B) are from the ARAS dataset and the third house is of the CASAS dataset. Both the datasets have sensor observations of two residents. The experiments are designed such that for all the three houses classification of activities of the residents are performed using different LSTM and BiLSTM networks and in each model, we explored oversampling, undersampling, and cost-sensitive learning methods to handle class imbalance problem. Each house of the ARAS dataset consists of 30 days of human activities data, where each day consists of 86,400 data points. The dataset is divided into training, validation and test set such that the first 18 days are used for training, the next six days of the dataset are used for validation and the last six days are used as a test set. In the CASAS-Kyoto Multiresident ADL activities dataset, human activities of two residents were carried out for 26 days and each file has a different number of data points. We followed a similar approach as other datasets, where the first 16 days are used as training (10572 instances), the next five days are used as validation test (3051 instances) and the last 5 days are used as test set (3608 instances of sensor readings). The experiments are computed first with the original dataset (without applying class imbalance methods) and then twelve different experiments are conducted for each model by applying class imbalance techniques to the training data.

The evaluation metrics play an important role in measuring the performance of models in handling class imbalance in multiple resident activity recognition. Hence, we used the Exact Match Ratio (EMR), Balanced accuracy, and micro average of F1-score to evaluate all the models. EMR metrics indicate the percentage of samples that have all their labels classified correctly (shown in Eq. 7). The balanced accuracy metric is used in multi-class classification problems to deal with imbalanced datasets and is based on two most commonly used metrics: sensitivity (also known as true positive rate or recall) and specificity (also known as a false-positive rate), shown in Eq. 8. Also, we used a micro average of F1-score as it is a weighted average of recall and precision, shown in Eq. 9. The Exact match ratio of both residents, balanced accuracy, and micro average of F1-score of each resident of the test set are computed at best validation accuracy for all the models.

In both LSTM and BiLSTM networks, we used a range of sequence lengths from 10 to 100, a range of batch sizes from 32 to 512, and a range of several epochs from 5 to 100. A series of trial and error experiments were conducted over these ranges. We observed that epochs = 30, batch size = 64, sequence length = 30, and hidden units (n) = 128 are found to be optimal parameters to avoid overfitting and achieved a low generalization error in training both the models. The model parameters are kept the same for all the datasets. The training of the network is performed on a single Quadro RTX 4000 8GB GPU, also trained models can be used for inference without losing much performance when there is no GPU. In addition, we also performed experiments on a single NVIDIA 12GB GeForce GTX 1080Ti GPU, and the same results were observed on both the computer environment.

7 $$\begin{aligned} Exact\,Match\,Ratio,EMR = \frac{1}{n}\sum _{i=1}^{n}I(Y_i=Z_i) \end{aligned}$$

where *I* is the indicator function, $Y_{i}$ is target class and $Z_{i}$ is predicted class.

8 $$\begin{aligned} Balanced\,Accuracy&= \frac{Sensitivity + Specificity}{2} \end{aligned}$$

9 $$\begin{aligned} F1-score&= \frac{2 * (precision * recall)}{(precision + recall)} \end{aligned}$$

## Results and discussion

**Fig. 5:**
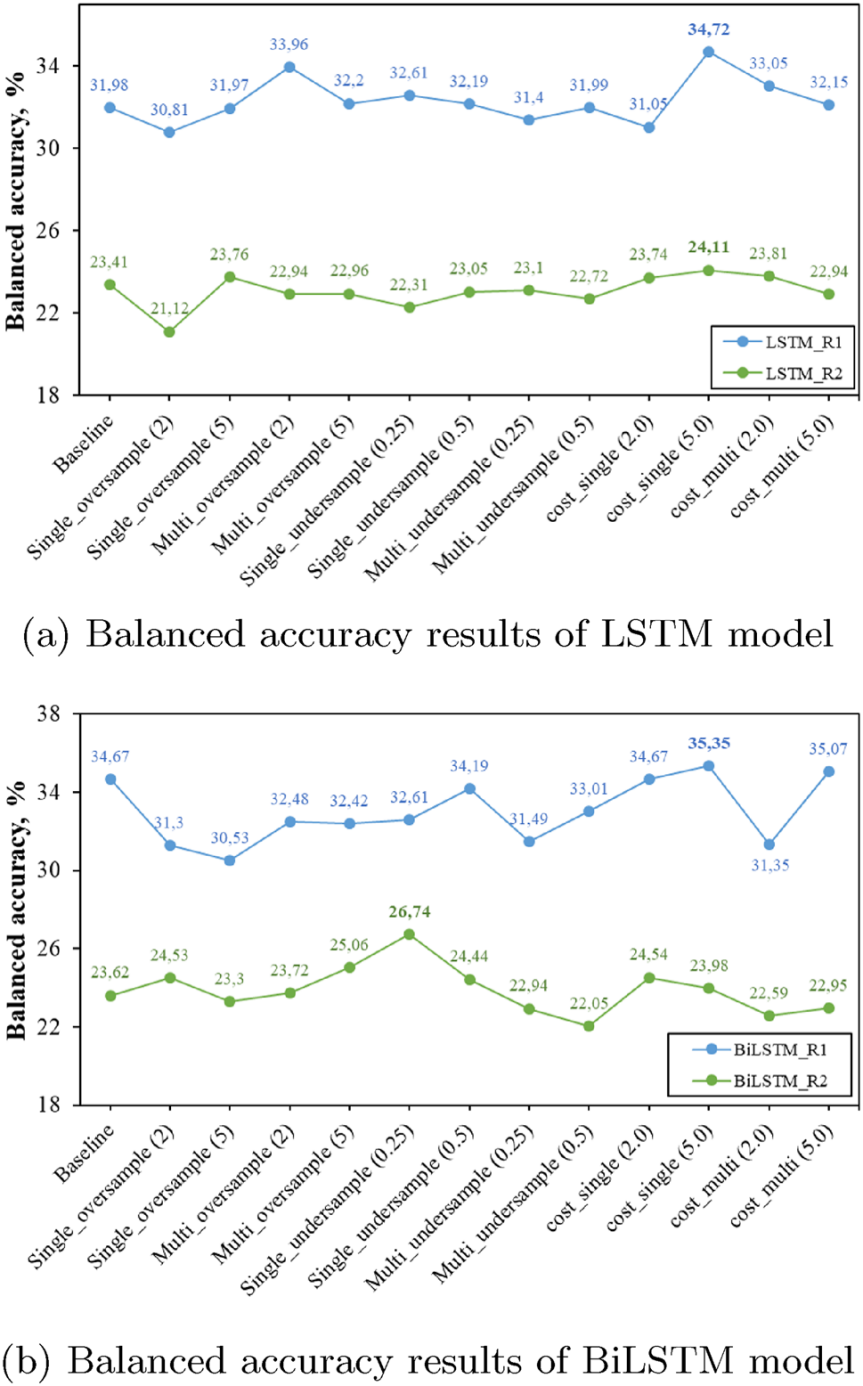
ARAS House A Balanced accuracy results

**Fig. 6:**
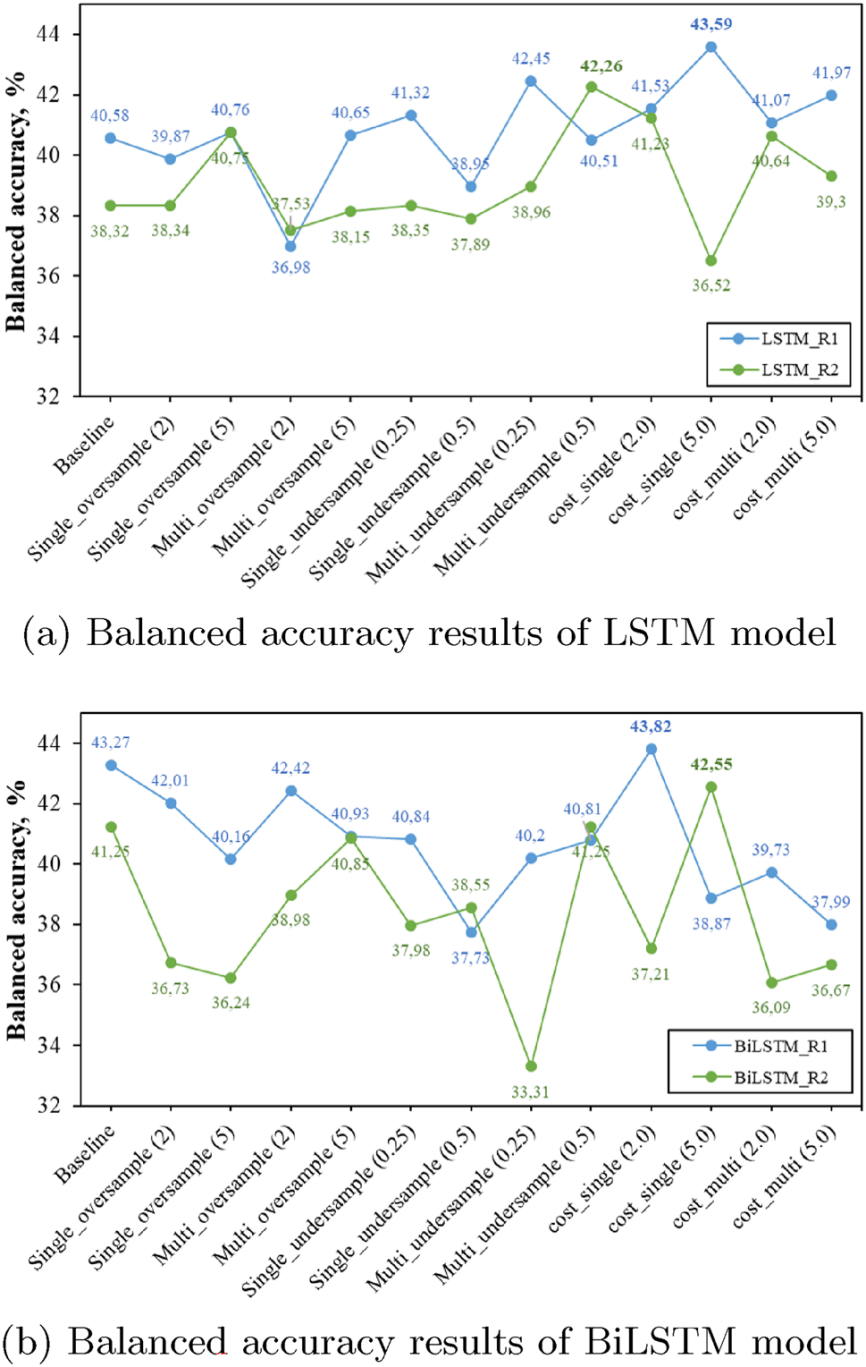
ARAS House B Balanced accuracy results

**Fig. 7:**
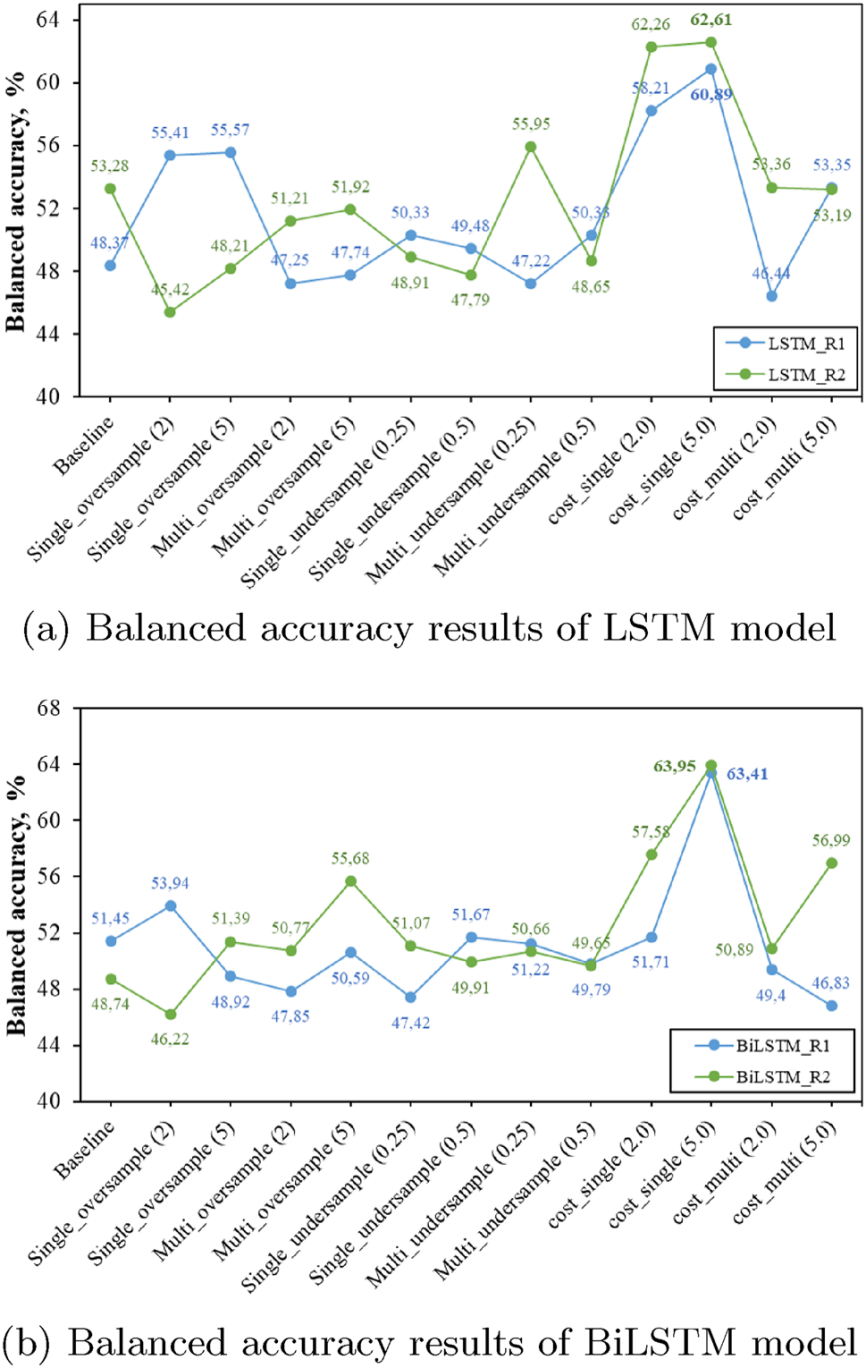
CASAS-Kyoto Balanced accuracy results

In this section, the experimental results of both LSTM and BiLSTM networks together with different class imbalance approaches in terms of exact match ratio, balanced accuracy, and a micro average of F1-score are presented and discussed. Figures 5, 6 and 7 present the balanced accuracy results of each resident of the dataset. Tables 1 and 2 report the results of House A (ARAS) dataset, Tables 3 and 4 report the results of House B (ARAS) and Tables 5 and 6 present the results of the CASAS-Kyoto Multiresident ADL Activities dataset in terms of EMR and micro average F1-score.

As discussed in the previous section, each table shows the experiment results of the baseline model which is without applying class imbalance techniques, and then 12 different experiment results with data level and algorithm level techniques on deep learning models. In all three approaches (oversample, undersample, and cost-sensitive learning), the term "single" represents activity recognition of each resident separately and the term "multi" represents combined activity recognition for the results. The models are evaluated at different oversample (2 and 5) and undersample (0.25 and 0.5) class ratios, together with different cost coefficient values (2 and 5) to have a detailed study and comparison of different class imbalance approaches in a multi-resident setting.

Balanced accuracy results show that a single cost-sensitive learning approach outperforms all the other class imbalance approaches in the majority of the cases. In the ARAS house A dataset, the single cost-sensitive learning approach of R1 improves by 3% in the LSTM and 1% in the BiLSTM in comparison to the baseline model, whereas in R2 cost-sensitive approach increases balanced accuracy by 1% in LSTM network but in BiLSTM model single-undersampling improves the balance accuracy by 3%. In House B of the ARAS dataset, the cost-sensitive approach performs better in both LSTM and BiLSTM models, except in the LSTM model of R2, where the undersampling approach is slightly better. In the CASAS dataset, a single cost-sensitive approach clearly outperforms all the other approaches and improves the balance accuracy results of R1 by 9% and 13% in LSTM and BiLSTM, 11% and 14% increase in balance accuracy of R2 LSTM and BiLSTM models in comparison to a baseline model.

**Table 1:** LSTM-HouseA (ARAS)

LSTM model	Both person (EMR) (%)	F1-score (R1)	F1-score (R2)
Baseline	54.03	0.65	0.73
Single_oversample (2)	52.46	0.64	0.72
Single_oversample (5)	50.52	0.60	0.73
Multi_oversample (2)	53.25	0.64	0.74
Multi_oversample (5)	51.50	0.63	0.72
Single_undersample (0.25)	52.87	0.65	0.73
Single_undersample (0.5)	53.15	0.64	0.73
Multi_undersample (0.25)	53.39	0.64	0.73
Multi_undersample (0.5)	52.26	0.64	0.73
Cost_single (2)	53.03	0.66	0.71
Cost_single (5)	51.14	0.63	0.72
Cost_multi (2)	52.80	0.64	0.73
Cost_multi (5)	52.85	0.64	0.73

**Table 2:** BiLSTM-HouseA (ARAS)

LSTM model	Both person (EMR) (%)	F1-score (R1)	F1-score (R2)
Baseline	53.89	0.65	0.74
Single_oversample (2)	52.76	0.64	0.74
Single_oversample (5)	50.89	0.62	0.72
Multi_oversample (2)	53.60	0.65	0.74
Multi_oversample (5)	51.59	0.63	0.72
Single_undersample (0.25)	53.96	0.65	0.74
Single_undersample (0.5)	53.57	0.65	0.73
Multi_undersample (0.25)	54.28	0.65	0.74
Multi_undersample (0.5)	53.41	0.65	0.72
Cost_single (2)	51.67	0.63	0.72
Cost_single (5)	50.44	0.64	0.68
Cost_multi (2)	54.06	0.65	0.74
Cost_multi (5)	53.80	0.66	0.74

To summarize, from the following results it can be observed that in almost all the networks cost-sensitive learning performs better in terms of balanced accuracy. In the EMR of both residents, no clear trend has been observed in the results, as in House B the difference in EMR results is minimal for both LSTM and BiLSTM networks, in House A, the baseline model performed better in comparison with other models, whereas in the BiLSTM network the results of EMR in undersampling and cost-sensitive approach are similar. In the CASAS-Kyoto dataset, EMR results are better in undersampling and cost-sensitive approach. The F1-score of R2 is better than R1 in the case of House A, whereas for House B high F1 score is achieved for both the residents in comparison to House A. Furthermore, in the CASAS-Kyoto smart home no significant difference can be seen in F1 scores of R1 and R2.

**Table 3:** LSTM-HouseB (ARAS)

LSTM model	Both person (EMR) (%)	F1-score (R1)	F1-score (R2)
Baseline	90.56	0.94	0.93
Single_oversample (2)	90.83	0.94	0.93
Single_oversample (5)	90.25	0.93	0.93
Multi_oversample (2)	90.44	0.94	0.93
Multi_oversample (5)	90.24	0.93	0.93
Single_undersample (0.25)	90.58	0.94	0.93
Single_undersample (0.5)	90.56	0.93	0.93
Multi_undersample (0.25)	90.75	0.94	0.93
Multi_undersample (0.5)	90.90	0.94	0.93
Cost_single (2)	90.83	0.93	0.93
Cost_single (5)	90.82	0.94	0.93
Cost_multi (2)	90.64	0.93	0.94
Cost_multi (5)	90.93	0.94	0.93

**Table 4:** BiLSTM-HouseB (ARAS)

LSTM model	Both person (EMR) (%)	F1-score (R1)	F1-score (R2)
Baseline	91.42	0.94	0.94
Single_oversample (2)	90.82	0.94	0.93
Single_oversample (5)	90.23	0.94	0.92
Multi_oversample (2)	91.18	0.95	0.93
Multi_oversample (5)	90.64	0.94	0.93
Single_undersample (0.25)	90.73	0.94	0.93
Single_undersample (0.5)	90.83	0.94	0.93
Multi_undersample (0.25)	90.82	0.94	0.93
Multi_undersample (0.5)	91.09	0.94	0.93
Cost_single (2)	91.29	0.94	0.93
Cost_single (5)	90.29	0.94	0.93
Cost_multi (2)	91.15	0.94	0.94
Cost_multi (5)	90.68	0.93	0.93

**Fig. 8:**
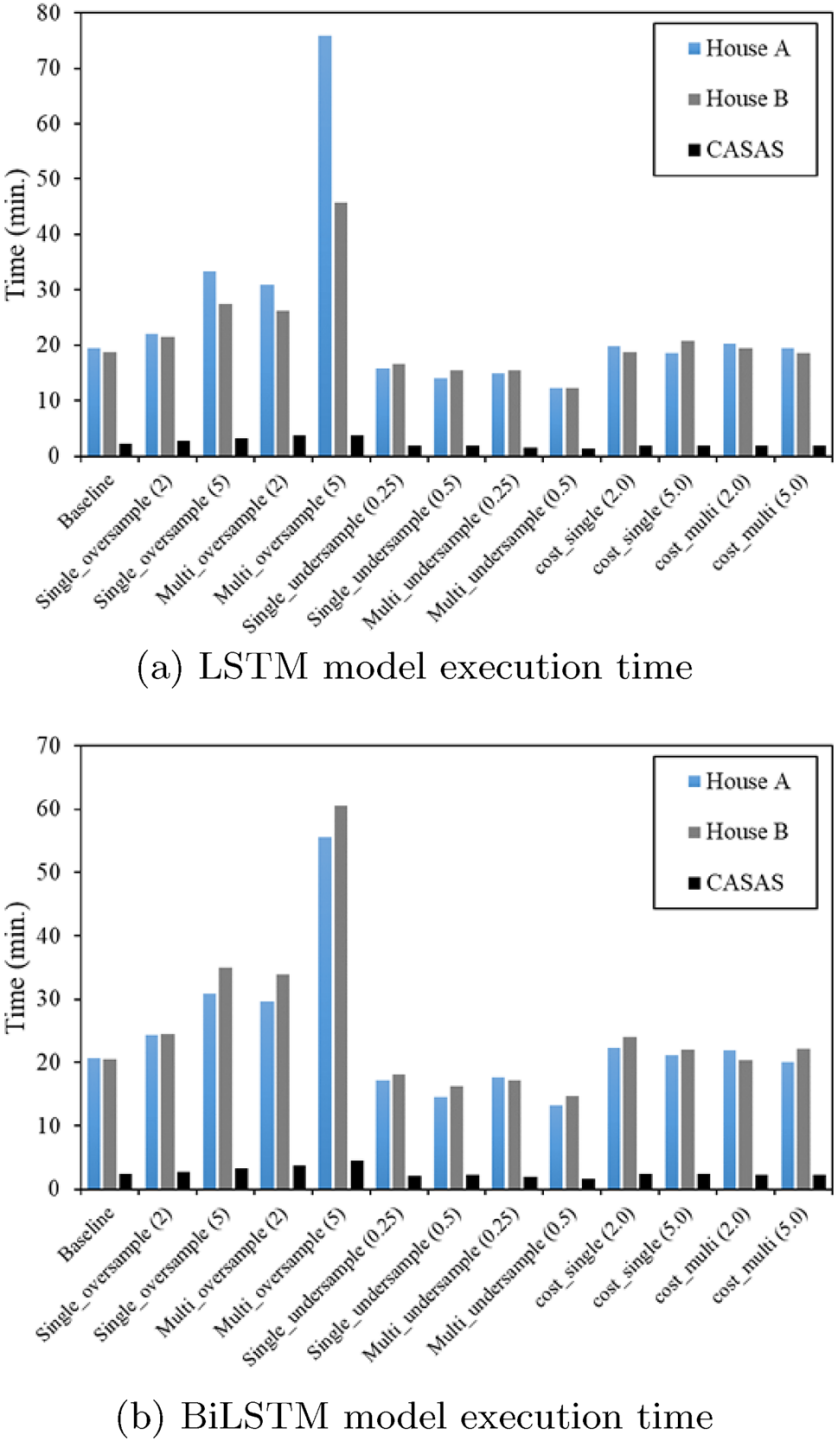
Model execution time

Since each SH dataset has a different configuration, sensor readings, activity labels, and class imbalance, the difference in model performance is observed in all three datasets. The computation time of the CASAS-Kyoto dataset was much faster in comparison to the ARAS dataset due to less number of sensor observations in each day of the dataset. In terms of model computational time, the undersampling method was faster to train in comparison to oversampling and cost-sensitive learning, where multi-oversampling took a quite long time to train which is quite obvious due to the increase in the number of samples to train the models. Among the deep learning models, training with LSTM was faster in comparison to the BiLSTM model. Figure 8 shows the computational time of both the models for all the three datasets.

**Table 5:** LSTM (CASAS-Kyoto)

LSTM model	Both person (EMR) (%)	F1-score (R1)	F1-score (R2)
Baseline	37.07	0.55	0.54
Single_oversample (2)	35.44	0.57	0.54
Single_oversample (5)	33.91	0.56	0.52
Multi_oversample (2)	35.88	0.57	0.54
Multi_oversample (5)	34.20	0.56	0.52
Single_undersample (0.25)	37.01	0.57	0.53
Single_undersample (0.5)	37.53	0.58	0.55
Multi_undersample (0.25)	38.86	0.58	0.54
Multi_undersample (0.5)	34.20	0.58	0.51
Cost_single (2)	35.16	0.54	0.53
Cost_single (5)	26.00	0.54	0.48
Cost_multi (2)	35.62	0.58	0.53
Cost_multi (5)	37.04	0.58	0.54

**Table 6:** BiLSTM (CASAS-Kyoto)

BiLSTM model	Both person (EMR) (%)	F1-score (R1)	F1-score (R1)
Baseline	37.64	0.57	0.55
Single_oversample (2)	36.52	0.58	0.54
Single_oversample (5)	34.89	0.58	0.53
Multi_oversample (2)	36.78	0.58	0.55
Multi_oversample (5)	34.08	0.59	0.53
Single_undersample (0.25)	35.82	0.58	0.53
Single_undersample (0.5)	37.85	0.57	0.54
Multi_undersample (0.25)	37.04	0.60	0.54
Multi_undersample (0.5)	35.82	0.59	0.54
Cost_single (2)	33.10	0.57	0.52
Cost_single (5)	24.32	0.53	0.51
Cost_multi (2)	35.21	0.58	0.53
Cost_multi (5)	32.11	0.57	0.52

### Results on frequent activities

In order to have a further comprehensive analysis of different class, imbalance approaches on multi-resident activity recognition datasets, we extended our experiments by selecting the first top-five activities of the dataset, and performed classifications using the same LSTM and BiLSTM as described above. Since even after oversampling and undersampling the data the distribution is still imbalance, we selected the top five activities in all the datasets to analyze model performance on frequent activities. The model configurations are exactly the same as previous experiments and the results of the experiments of ARAS are shown in Tables 7, 8, 9 and 10. The model configurations in the CASAS frequent activities dataset are also the same as previous CASAS experiments. Tables 11 and 12 present the results of class imbalance techniques on frequent activities of the CASAS-Kyoto dataset.

**Table 7:** LSTM-House A (ARAS)

LSTM model	Both person (EMR) (%)	Balanced accuracy (R1) (%)	Balanced accuracy (R2) (%)	F1-score (R1)	F1-score (R2)
Baseline	77.95	75.02	60.13	0.87	0.86
Single_oversample (2)	74.94	73.63	58.43	0.84	0.85
Single_oversample (5)	75.68	71.58	61.47	0.86	0.84
Multi_oversample (2)	78.09	74.90	60.27	0.87	0.86
Multi_oversample (5)	75.29	74.64	57.05	0.85	0.84
Single_undersample (0.25)	78.76	73.07	62.29	0.86	0.86
Single_undersample (0.5)	75.86	73.66	60.83	0.86	0.85
Multi_undersample (0.25)	77.13	71.96	61.94	0.86	0.85
Multi_undersample (0.5)	76.21	73.15	61.96	0.85	0.85
Cost_single (2)	75.58	73.17	62.97	0.85	0.84
Cost_single (5)	73.68	73.82	57.86	0.84	0.82
Cost_multi (2)	78.11	73.27	64.27	0.87	0.87
Cost_multi (5)	76.62	73.42	62.67	0.85	0.85

**Table 8:** BiLSTM-House A (ARAS)

BiLSTM model	Both person (EMR) (%)	Balanced accuracy (R1) (%)	Balanced accuracy (R2) (%)	F1-score (R1)	F1-score (R2)
Baseline	78.58	73.02	64.71	0.87	0.87
Single_oversample (2)	77.95	72.51	65.19	0.87	0.88
Single_oversample (5)	75.64	74.61	59.92	0.86	0.84
Multi_oversample (2)	79.09	75.30	65.31	0.88	0.88
Multi_oversample (5)	75.49	72.25	62.72	0.84	0.86
Single_undersample (0.25)	79.01	74.46	60.81	0.88	0.87
Single_undersample (0.5)	79.35	75.22	64.12	0.87	0.88
Multi_undersample (0.25)	77.37	71.13	65.19	0.85	0.87
Multi_undersample (0.5)	78.05	74.18	64.19	0.87	0.86
Cost_single (2)	76.48	75.88	62.08	0.87	0.84
Cost_single (5)	74.23	72.71	62.47	0.86	0.82
Cost_multi (2)	78.27	74.02	63.52	0.88	0.88
Cost_multi (5)	77.01	74.73	65.96	0.85	0.88

**Table 9:** LSTM-House B (ARAS)

LSTM model	Both person (EMR) (%)	Balanced accuracy (R1) (%)	Balanced accuracy (R2) (%)	F1-score (R1)	F1-score (R2)
Baseline	96.73	82.14	88.07	0.98	0.98
Single_oversample (2)	96.98	83.48	91.80	0.98	0.98
Single_oversample (5)	96.46	81.66	87.94	0.98	0.98
Multi_oversample (2)	96.55	80.42	88.43	0.97	0.98
Multi_oversample (5)	96.41	83.72	85.03	0.98	0.97
Single_undersample (0.25)	97.14	77.90	91.07	0.98	0.98
Single_undersample (0.5)	97.07	81.54	84.37	0.98	0.98
Multi_undersample (0.25)	96.75	83.66	90.37	0.97	0.99
Multi_undersample (0.5)	96.97	83.53	86.51	0.98	0.99
Cost_single (2)	97.23	83.07	91.02	0.98	0.98
Cost_single (5)	96.51	82.51	81.22	0.97	0.98
Cost_multi (2)	96.38	80.51	84.69	0.98	0.98
Cost_multi (5)	96.49	76.98	86.75	0.97	0.98

**Table 10:** BiLSTM-House B (ARAS)

BiLSTM model	Both person (EMR) (%)	Balanced accuracy (R1) (%)	Balanced accuracy (R2) (%)	F1-score (R1)	F1-score (R2)
Baseline	96.88	84.98	**93.88**	0.98	0.98
Single_oversample (2)	96.79	84.21	93.14	0.98	0.98
Single_oversample (5)	97.06	83.85	87.47	0.98	0.98
Multi_oversample (2)	96.83	81.08	90.61	0.98	0.98
Multi_oversample (5)	96.45	84.14	83.19	0.97	0.99
Single_undersample (0.25)	97.23	84.56	83.96	0.98	0.99
Single_undersample (0.5)	97.31	83.10	92.67	0.98	0.99
Multi_undersample (0.25)	97.85	85.68	90.76	0.98	0.99
Multi_undersample (0.5)	96.95	80.78	83.73	0.98	0.98
Cost_single (2)	97.35	83.40	90.82	0.98	0.98
Cost_single (5)	96.91	86.67	81.27	0.98	0.98
Cost_multi (2)	96.91	80.21	88.02	0.97	0.99
Cost_multi (5)	96.89	82.16	92.60	0.98	0.99

**Table 11:** LSTM-(CASAS-Kyoto)

LSTM model	Both person (EMR) (%)	Balanced accuracy (R1) (%)	Balanced accuracy (R2) (%)	F1-score (R1)	F1-score (R1)
Baseline	22.87	27.01	38.19	0.36	0.41
Single_oversample (2)	19.49	25.17	25.17	0.36	0.39
Single_oversample (5)	24.66	30.30	44.51	0.34	0.41
Multi_oversample (2)	23.97	27.74	33.41	0.42	0.32
Multi_oversample (5)	25.41	30.04	37.59	0.35	0.44
Single_undersample (0.25)	20.45	24.18	30.81	0.25	0.45
Single_undersample (0.5)	24.45	27.71	30.85	0.30	0.40
Multi_undersample (0.25)	27.07	27.38	36.48	0.36	0.46
Multi_undersample (0.5)	18.46	30.10	35.47	0.29	0.44
Cost_single (2)	25.14	30.48	34.86	0.36	0.32
Cost_single (5)	22.59	29.63	38.53	0.33	0.41
Cost_multi (2)	22.11	27.77	45.75	0.36	0.42
Cost_multi (5)	26.72	26.71	44.59	0.38	0.41

**Table 12:** BiLSTM-(CASAS-Kyoto)

BiLSTM model	Both person (EMR) (%)	Balanced accuracy (R1) (%)	Balanced accuracy (R2) (%)	F1-score (R1)	F1-score (R1)
Baseline	22.52	25.23	33.89	0.32	0.41
Single_oversample (2)	26.65	24.80	46.07	0.35	0.46
Single_oversample (5)	21.42	20.54	42.72	0.27	0.43
Multi_oversample (2)	29.27	31.47	40.81	0.40	0.40
Multi_oversample (5)	21.28	25.55	29.76	0.33	0.37
Single_undersample (0.25)	21.28	23.94	36.21	0.29	0.43
Single_undersample (0.5)	21.14	20.71	36.93	0.34	0.36
Multi_undersample (0.25)	25.00	32.58	31.00	0.38	0.39
Multi_undersample (0.5)	26.93	32.21	47.48	0.36	0.46
Cost_single (2)	24.17	29.72	36.98	0.28	0.44
Cost_single (5)	22.31	26.21	37.61	0.33	0.39
Cost_multi (2)	28.24	30.06	37.65	0.36	0.38
Cost_multi (5)	27.75	30.03	43.14	0.36	0.46

The EMR, balanced accuracy, and F1 score of both House A and House B of the ARAS dataset improved a lot in comparison to previous experiments when we took frequent activities of the datasets, which also makes the dataset quite balanced and thus improving the performance of LSTM and BiLSTM models. The results of EMR improved a lot in comparison to previous experiments but are similar in each dataset for all the approaches. In the balanced accuracy results of frequent activities, again cost-sensitive approach performed better in most of the cases in comparison to oversampling and undersampling methods. There were few cases such as in House B, in R2 activities classification, the oversampling approach in LSTM, and the baseline model of the BiLSTM network performed better than other approaches. However, the cost-sensitive approach performed equally in these cases, for example, the results of cost single (2) and single oversample (2) are almost equal in the LSTM model. Similarly, in the BiLSTM network of House B, the difference between baseline and cost-multi (5) is much less. In the CASAS-Kyoto dataset, the multi-undersampling method performed better in the BiLSTM network for both residents. However, in per class F1-score results, the cost-sensitive method performed better in the classification of minority classes.

The CASAS-Kyoto dataset showed improvement in class imbalance techniques (Tables 11, 12) in comparison to baseline models such as in the LSTM model of CASAS. The cost-sensitive method outperformed all the other methods and in the BiLSTM model of CASAS, undersampling approach performed better, but the results of F1-score of the cost-sensitive approach are almost similar to the undersampling method for each class. The micro average F1-score of both House A and House B improved a lot in frequent activity experiments, whereas in CASAS it did not show much improvement. This can be due to the "curse of dimensionality" in the SH datasets, as not all sensors are relevant to the classification and high dimensions deteriorate the performance of the classifier. Furthermore, it has been observed that the CASAS-Kyoto dataset showed a difference in the performance of the model with different class imbalance techniques, whereas in the ARAS dataset not much clear trend is observed. This can be attributed to the fact that the CASAS-Kyoto dataset is quite a balanced dataset whereas the ARAS dataset is highly imbalanced.

## Conclusion

In the realm of multiple resident activity recognition, which is integral to the enhancement of smart technologies [75], elder care [76], and ambient assisted living systems [77], as well as safety and context-aware applications [78], the importance of explainability, retraceability, and human interpretability cannot be overstated. Explainability is paramount in the application of complex models such as LSTM and BiLSTM networks, as it fosters trust and acceptance among users and stakeholders. The ability to interpret model decisions is critical, especially in environments such as health care and ambient assisted living, where decisions must be transparent and justifiable. This study, through the lens of class imbalance techniques, has not only sought to enhance the accuracy of activity recognition systems but also contributed to the field of explainable AI by exploring how different techniques can influence model interpretability. Retraceability, the ability to audit the data and processes that lead to a particular model decision, is essential for compliance with regulatory frameworks that govern AI systems, particularly in Europe where the right to explanation is an emerging requirement [79]. By meticulously documenting the experimental setup, including data processing, model configuration, and the application of class imbalance techniques, this study provides a blueprint for retraceability. Human interpretability is inherently linked with the first two concepts, emphasizing the need for model predictions to be understandable by humans. This is especially pertinent when AI systems are used to support decision-making in critical settings. The study’s findings suggest that cost-sensitive learning can improve performance metrics, such as balanced accuracy, which is a step toward making AI decisions more interpretable. The interpretability of such approaches must be further investigated to ensure that users can comprehend and trust the system’s predictions. The discussion on scalability also implicitly acknowledges the challenges of maintaining explainability and interpretability as the complexity of the environment increases. With more residents and potentially more complex class distributions, the importance of designing AI systems that are not only accurate but also explainable and interpretable becomes more pronounced. Thus, this research does not merely present a set of algorithms for activity recognition but also paves the way for future studies that must consider these critical dimensions of AI development in order to be deemed trustworthy and human-centered.

To elevate the performance of our model, particularly concerning the minority class, future endeavors will be directed towards the examination of alternative deep learning architectures and hybrid methodologies that are adept at negotiating class imbalance within the multi-resident milieu [80]. In the pursuit of universal access in the information society, it is critical to align these technological advances with the principles of explainable AI, especially in the context of graph neural networks [81, 82]. The ambition is to construct models that are not only effective but also human-interpretable, particularly for temporal Smart Home (SH) datasets.

Human interpretability engenders an understanding of the reasoning behind network decisions, which in turn cultivates trust in the system-a necessity for the universal adoption of such technologies [83]. Furthermore, the deployment of AI in settings with diverse and potentially vulnerable populations accentuates the need for transparent and accountable systems.

As we forge ahead, it is imperative to recognize that novel evaluation paradigms are required to adequately assess the efficacy of such models within the context of imbalanced datasets [84]. These new paradigms must address not only the technical accuracy of model predictions but also the explainability and fairness [85] of these predictions to ensure that AI systems contribute positively to the inclusivity and accessibility of the information society. Thus, our future research is poised to contribute to this critical discourse, ensuring that the advancements in AI are both technically sound and ethically responsible, facilitating a more equitable information society.

## References

[1] Ranasinghe, S., Al Machot, F., Mayr, H.C.: A review on applications of activity recognition systems with regard to performance and evaluation. Int. J. Distrib. Sens. Netw. **12**(8), 1550147716665520 (2016)

[2] Singh, D., Psychoula, I., Merdivan, E., Kropf, J., Hanke, S., Sandner, E., Chen, L., Holzinger, A.: Privacy-enabled smart home framework with voice assistant. Smart Assisted Living: Toward An Open Smart-Home Infrastructure, 321–339 (2020)

[3] Palumbo, F., Gallicchio, C., Pucci, R., Micheli, A.: Human activity recognition using multisensor data fusion based on reservoir computing. J. Ambient Intell. Smart Environ. **8**(2), 87–107 (2016)

[4] Singh, D., Merdivan, E., Psychoula, I., Kropf, J., Hanke, S., Geist, M., Holzinger, A.: Human activity recognition using recurrent neural networks. In: International Cross-Domain Conference for Machine Learning and Knowledge Extraction, pp. 267–274 (2017). Springer

[5] Hoque, E., Stankovic, J.: Aalo: Activity recognition in smart homes using active learning in the presence of overlapped activities. In: 2012 6th International Conference on Pervasive Computing Technologies for Healthcare (PervasiveHealth) and Workshops, pp. 139–146 (2012). IEEE

[6] Chen, L., Nugent, C.D., Wang, H.: A knowledge-driven approach to activity recognition in smart homes. IEEE Trans. Knowl. Data Eng. **24**(6), 961–974 (2011)

[7] Rafferty, J., Nugent, C.D., Liu, J., Chen, L.: From activity recognition to intention recognition for assisted living within smart homes. IEEE Trans. Hum.-Mach. Syst. **47**(3), 368–379 (2017)

[8] Benmansour, A., Bouchachia, A., Feham, M.: Multioccupant activity recognition in pervasive smart home environments. ACM Comput. Surv. (CSUR) **48**(3), 34 (2016)

[9] FernáNdez, A., LóPez, V., Galar, M., Del Jesus, M.J., Herrera, F.: Analysing the classification of imbalanced data-sets with multiple classes: binarization techniques and ad-hoc approaches. Knowl.-Based Syst. **42**, 97–110 (2013)

[10] Thai-Nghe, N., Gantner, Z., Schmidt-Thieme, L.: Cost-sensitive learning methods for imbalanced data. In: The 2010 International Joint Conference on Neural Networks (IJCNN), pp. 1–8 (2010). IEEE

[11] Singh, D., Merdivan, E., Hanke, S., Kropf, J., Geist, M., Holzinger, A.: Convolutional and recurrent neural networks for activity recognition in smart environment. In: Towards Integrative Machine Learning and Knowledge Extraction: BIRS Workshop, Banff, AB, Canada, July 24-26, 2015, Revised Selected Papers, pp. 194–205 (2017). Springer

[12] Nguyen, N., Venkatesh, S., Bui, H.: Recognising behaviours of multiple people with hierarchical probabilistic model and statistical data association. In: BMVC 2006: Proceedings of the 17th British Machine Vision Conference, pp. 1239–1248 (2006). British Machine Vision Association

[13] Du, Y., Chen, F., Xu, W.: Human interaction representation and recognition through motion decomposition. IEEE Signal Process. Lett. **14**(12), 952–955 (2007)

[14] Natarajan, P., Nevatia, R.: Coupled hidden semi markov models for activity recognition. In: 2007 IEEE Workshop on Motion and Video Computing (WMVC’07), pp. 10–10 (2007). IEEE

[15] Crandall, A.S., Cook, D.J.: Using a hidden markov model for resident identification. In: 2010 Sixth International Conference on Intelligent Environments, pp. 74–79 (2010). IEEE

[16] Singla, G., Cook, D.J., Schmitter-Edgecombe, M.: Recognizing independent and joint activities among multiple residents in smart environments. J. Ambient. Intell. Humaniz. Comput. **1**(1), 57–63 (2010) 20975986 10.1007/s12652-009-0007-1 PMC2958106

[17] Wang, L., Gu, T., Tao, X., Chen, H., Lu, J.: Recognizing multi-user activities using wearable sensors in a smart home. Pervasive Mob. Comput. **7**(3), 287–298 (2011)

[18] Singh, D., Kropf, J., Hanke, S., Holzinger, A.: Ambient assisted living technologies from the perspectives of older people and professionals. In: International Cross-Domain Conference for Machine Learning and Knowledge Extraction, pp. 255–266 (2017). Springer

[19] Singh, D., Psychoula, I., Kropf, J., Hanke, S., Holzinger, A.: Users’ perceptions and attitudes towards smart home technologies. In: International Conference on Smart Homes and Health Telematics, pp. 203–214 (2018). Springer

[20] Fu, B., Damer, N., Kirchbuchner, F., Kuijper, A.: Sensing technology for human activity recognition: A comprehensive survey. Ieee Access **8**, 83791–83820 (2020). 10.1109/ACCESS.2020.2991891

[21] Gravina, R., Alinia, P., Ghasemzadeh, H., Fortino, G.: Multi-sensor fusion in body sensor networks: state-of-the-art and research challenges. Inf. Fusion **35**, 68–80 (2017)

[22] Qin, Z., Zhang, Y., Meng, S., Qin, Z., Choo, K.-K.R.: Imaging and fusing time series for wearable sensor-based human activity recognition. Inf. Fusion **53**, 80–87 (2020)

[23] Machado, E., Singh, D., Cruciani, F., Chen, L., Hanke, S., Salvago, F., Kropf, J., Holzinger, A.: A conceptual framework for adaptive user interfaces for older adults. In: 2018 IEEE International Conference on Pervasive Computing and Communications Workshops (PerCom Workshops), pp. 782–787 (2018). IEEE

[24] Stephanidis, C.: Adaptive techniques for universal access. User Model. User-Adap. Inter. **11**, 159–179 (2001)

[25] Stephanidis, C., Savidis, A.: Universal access in the information society: methods, tools, and interaction technologies. Univ. Access Inf. Soc. **1**, 40–55 (2001)

[26] Röcker, C., Ziefle, M., Holzinger, A.: From computer innovation to human integration: Current trends and challenges for pervasive health technologies. In: Pervasive Health: State-of-the-Art and Beyond, pp. 1–17. Springer, New York (2014). 10.1007/978-1-4471-6413-5_1

[27] Zainudin, M.S., Sulaiman, M.N., Mustapha, N., Perumal, T.: Activity recognition based on accelerometer sensor using combinational classifiers. In: 2015 Ieee Conference on Open Systems (Icos), pp. 68–73 (2015). IEEE

[28] Lu, C.-H., Fu, L.-C.: Robust location-aware activity recognition using wireless sensor network in an attentive home. IEEE Trans. Autom. Sci. Eng. **6**(4), 598–609 (2009)

[29] Crandall, A.S., Cook, D.J.: Resident and caregiver: Handling multiple people in a smart care facility. In: AAAI Fall Symposium: AI in Eldercare: New Solutions to Old Problems, pp. 39–47 (2008)

[30] Hsu, K.-C., Chiang, Y.-T., Lin, G.-Y., Lu, C.-H., Hsu, J.Y.-J., Fu, L.-C.: Strategies for inference mechanism of conditional random fields for multiple-resident activity recognition in a smart home. In: International Conference on Industrial, Engineering and Other Applications of Applied Intelligent Systems, pp. 417–426 (2010). Springer

[31] Cook, D., Schmitter-Edgecombe, M., Crandall, A., Sanders, C., Thomas, B.: Collecting and disseminating smart home sensor data in the casas project. In: Proceedings of the CHI Workshop on Developing Shared Home Behavior Datasets to Advance HCI and Ubiquitous Computing Research, pp. 1–7 (2009)

[32] Chen, R., Tong, Y.: A two-stage method for solving multi-resident activity recognition in smart environments. Entropy **16**(4), 2184–2203 (2014)

[33] Liciotti, D., Bernardini, M., Romeo, L., Frontoni, E.: A sequential deep learning application for recognising human activities in smart homes. Neurocomputing (2019)

[34] Zhao, Y., Yang, R., Chevalier, G., Xu, X., Zhang, Z.: Deep residual bidir-lstm for human activity recognition using wearable sensors. Math. Probl. Eng. **2018** (2018)

[35] Li, X., Zhang, Y., Zhang, J., Chen, S., Marsic, I., Farneth, R.A., Burd, R.S.: Concurrent activity recognition with multimodal cnn-lstm structure. arXiv preprint arXiv:1702.01638 (2017)

[36] Hamad, R.A., Yang, L., Woo, W.L., Wei, B.: Joint learning of temporal models to handle imbalanced data for human activity recognition. Appl. Sci. **10**(15), 5293 (2020)

[37] Krawczyk, B.: Learning from imbalanced data: open challenges and future directions. Prog. Artif. Intell. **5**(4), 221–232 (2016)

[38] Hassler, A.P., Menasalvas, E., Garcia-Garcia, F.J., Rodriguez-Manas, L., Holzinger, A.: Importance of medical data preprocessing in predictive modeling and risk factor discovery for the frailty syndrome. Springer/Nat. BMC Med. Inform. Decis. Mak. **19**(1), 1–17 (2019). 10.1186/s12911-019-0747-6 10.1186/s12911-019-0747-6 PMC6483150 30777059

[39] Chawla, N.V., Bowyer, K.W., Hall, L.O., Kegelmeyer, W.P.: Smote: synthetic minority over-sampling technique. J. Artif. Intell. Res. **16**, 321–357 (2002)

[40] Fernandez, A., Garcia, S., Herrera, F.: Addressing the classification with imbalanced data: open problems and new challenges on class distribution. In: International Conference on Hybrid Artificial Intelligence Systems, pp. 1–10 (2011). Springer

[41] Mani, I., Zhang, I.: knn approach to unbalanced data distributions: a case study involving information extraction. In: Proceedings of Workshop on Learning from Imbalanced Datasets, vol. 126 (2003)

[42] Zhou, Z.-H., Liu, X.-Y.: On multi-class cost-sensitive learning. Comput. Intell. **26**(3), 232–257 (2010)

[43] Woźniak, M., Graña, M., Corchado, E.: A survey of multiple classifier systems as hybrid systems. Inf. Fusion **16**, 3–17 (2014)

[44] Wang, S., Li, Z., Chao, W., Cao, Q.: Applying adaptive over-sampling technique based on data density and cost-sensitive svm to imbalanced learning. In: The 2012 International Joint Conference on Neural Networks (IJCNN), pp. 1–8 (2012). IEEE

[45] Lin, M., Tang, K., Yao, X.: Dynamic sampling approach to training neural networks for multiclass imbalance classification. IEEE Trans. Neural Netw. Learn. Syst. **24**(4), 647–660 (2013) 24808384 10.1109/TNNLS.2012.2228231

[46] Krawczyk, B., Woźniak, M.: Cost-sensitive neural network with roc-based moving threshold for imbalanced classification. In: International Conference on Intelligent Data Engineering and Automated Learning, pp. 45–52 (2015). Springer

[47] Chawla, N.V., Cieslak, D.A., Hall, L.O., Joshi, A.: Automatically countering imbalance and its empirical relationship to cost. Data Min. Knowl. Disc. **17**(2), 225–252 (2008)

[48] Chathuramali, K.M., Rodrigo, R.: Faster human activity recognition with svm. In: International Conference on Advances in ICT for Emerging Regions (ICTer2012), pp. 197–203 (2012). IEEE

[49] Huang, C., Li, Y., Change Loy, C., Tang, X.: Learning deep representation for imbalanced classification. In: Proceedings of the IEEE Conference on Computer Vision and Pattern Recognition, pp. 5375–5384 (2016)

[50] Ando, S., Huang, C.Y.: Deep over-sampling framework for classifying imbalanced data. In: Joint European Conference on Machine Learning and Knowledge Discovery in Databases, pp. 770–785 (2017). Springer

[51] Wang, S., Liu, W., Wu, J., Cao, L., Meng, Q., Kennedy, P.J.: Training deep neural networks on imbalanced data sets. In: 2016 International Joint Conference on Neural Networks (IJCNN), pp. 4368–4374 (2016). IEEE

[52] Khan, S.H., Hayat, M., Bennamoun, M., Sohel, F.A., Togneri, R.: Cost-sensitive learning of deep feature representations from imbalanced data. IEEE Trans. Neural Netw. Learn. Syst. **29**(8), 3573–3587 (2017) 28829320 10.1109/TNNLS.2017.2732482

[53] Lin, E., Chen, Q., Qi, X.: Deep reinforcement learning for imbalanced classification. Appl. Intell. 1–15 (2020)

[54] Johnson, J.M., Khoshgoftaar, T.M.: Survey on deep learning with class imbalance. J. Big Data **6**(1), 27 (2019)

[55] Retzlaff, C.O., Das, S., Wayllace, C., Mousavi, P., Afshari, M., Yang, T., Saranti, A., Angerschmid, A., Taylor, M.E., Holzinger, A.: Human-in-the-loop reinforcement learning: a survey and position on requirements, challenges, and opportunities. J. Artif. Intell. Res. (JAIR) **79**(1), 349–415 (2024). 10.1613/jair.1.15348

[56] Schnake, T., Eberle, O., Lederer, J., Nakajima, S., Schütt, K.T., Müller, K.-R., Montavon, G.: Higher-order explanations of graph neural networks via relevant walks. IEEE Trans. Pattern Anal. Mach. Intell. **44**(11), 7581–7596 (2021). 10.1109/TPAMI.2021.3115452 10.1109/TPAMI.2021.3115452 34559639

[57] Lundberg, S.M., Lee, S.-I.: A unified approach to interpreting model predictions. Adv. Neural Inf. Process. Syst. **30**, (2017)

[58] Ribeiro, M.T., Singh, S., Guestrin, C.: “why should i trust you?” explaining the predictions of any classifier. In: Proceedings of the 22nd ACM SIGKDD International Conference on Knowledge Discovery and Data Mining, pp. 1135–1144 (2016)

[59] Holzinger, A., Goebel, R., Fong, R., Moon, T., Müller, K.-R., Samek, W.: xxai-beyond explainable artificial intelligence. In: International Workshop on Extending Explainable AI Beyond Deep Models and Classifiers, pp. 3–10 (2020). Springer

[60] Retzlaff, C.O., Angerschmid, A., Saranti, A., Schneeberger, D., Roettger, R., Mueller, H., Holzinger, A.: Post-hoc vs ante-hoc explanations: Xai design guidelines for data scientists. Cogn. Syst. Res. **86**(8), 101243 (2024). 10.1016/j.cogsys.2024.101243

[61] Cabitza, F., Campagner, A., Malgieri, G., Natali, C., Schneeberger, D., Stoeger, K., Holzinger, A.: Quod erat demonstrandum?-towards a typology of the concept of explanation for the design of explainable ai. Expert Syst. Appl. **213**(3), 1–16 (2023). 10.1016/j.eswa.2022.118888

[62] Kieseberg, P., Weippl, E., Tjoa, A.M., Cabitza, F., Campagner, A., Holzinger, A.: Controllable ai-an alternative to trustworthiness in complex ai systems? In: International Cross-Domain Conference for Machine Learning and Knowledge Extraction, pp. 1–12 (2023). Springer

[63] Dablain, D.A., Bellinger, C., Krawczyk, B., Aha, D.W., Chawla, N.V.: Understanding imbalanced data: Xai & interpretable ml framework

[64] Patil, A., Framewala, A., Kazi, F.: Explainability of smote based oversampling for imbalanced dataset problems. In: 2020 3rd International Conference on Information and Computer Technologies (ICICT), pp. 41–45 (2020). IEEE

[65] Slack, D., Hilgard, A., Singh, S., Lakkaraju, H.: Reliable post hoc explanations: modeling uncertainty in explainability. Adv. Neural. Inf. Process. Syst. **34**, 9391–9404 (2021)

[66] Zafar, M.R., Khan, N.M.: Dlime: A deterministic local interpretable model-agnostic explanations approach for computer-aided diagnosis systems. arXiv preprint arXiv:1906.10263 (2019)

[67] Chen, Y., Calabrese, R., Martin-Barragan, B.: Interpretable machine learning for imbalanced credit scoring datasets. Eur. J. Oper. Res. **312**(1), 357–372 (2024)

[68] Guo, S., Liu, Y., Chen, R., Sun, X., Wang, X.: Improved smote algorithm to deal with imbalanced activity classes in smart homes. Neural Process. Lett. **50**(2), 1503–1526 (2019)

[69] Oussalah, M., Hessami, A., Abidine, B.M., Fergani, B., Fergani, L.: A new classification strategy for human activity recognition using cost sensitive support vector machines for imbalanced data. Kybernetes (2014)

[70] Alemdar, H., Ertan, H., Incel, O.D., Ersoy, C.: Aras human activity datasets in multiple homes with multiple residents. In: 2013 7th International Conference on Pervasive Computing Technologies for Healthcare and Workshops, pp. 232–235 (2013). IEEE

[71] Cook, D.: Center of Advanced Studies in Adaptive System (CASAS). (2009 (last accessed April, 25, 2024)). https://casas.wsu.edu/datasets/

[72] Hochreiter, S., Schmidhuber, J.: Long short-term memory. Neural Comput. **9**(8), 1735–1780 (1997) 9377276 10.1162/neco.1997.9.8.1735

[73] Olah, C.: Understanding LSTM Networks. Accessed: 2020-11-10 (2015). https://colah.github.io/posts/2015-08-Understanding-LSTMs/

[74] Galar, M., Fernandez, A., Barrenechea, E., Bustince, H., Herrera, F.: A review on ensembles for the class imbalance problem: bagging-, boosting-, and hybrid-based approaches. IEEE Trans. Syst. Man Cybern. Part C Appl. Rev. **42**(4), 463–484 (2011)

[75] Augusto, J.C., Kramer, D., Alegre, U., Covaci, A., Santokhee, A.: The user-centred intelligent environments development process as a guide to co-create smart technology for people with special needs. Univ. Access Inf. Soc. **17**, 115–130 (2018). 10.1007/s10209-016-0514-8

[76] Zhou, J., Tan, R., Lin, H.-C.: Development of an integrated conceptual path model for a smart elderly care information system. Univ. Access Inf. Soc. **22**(3), 785–810 (2023). 10.1007/s10209-022-00879-7

[77] Caballero, P., Ortiz, G., Medina-Bulo, I.: Systematic literature review of ambient assisted living systems supported by the internet of things. Universal Access in the Information Society, 1–26 (2023) 10.1007/s10209-023-01022-w

[78] Stephanidis, C.: Designing for all in ambient intelligence environments: the interplay of user, context, and technology. Int. J. Hum.-Comput. Interact. **25**(5), 441–454 (2009). 10.1080/10447310902865032

[79] Stoeger, K., Schneeberger, D., Holzinger, A.: Medical artificial intelligence: the european legal perspective. Commun. ACM **64**(11), 34–36 (2021). 10.1145/3458652

[80] Bacciu, D., Errica, F., Micheli, A., Podda, M.: A gentle introduction to deep learning for graphs. Neural Networks (2020) 10.1016/j.neunet.2020.06.006 32559609

[81] Holzinger, A., Dehmer, M., Emmert-Streib, F., Cucchiara, R., Augenstein, I., Del Ser, J., Samek, W., Jurisica, I., Díaz-Rodríguez, N.: Information fusion as an integrative cross-cutting enabler to achieve robust, explainable, and trustworthy medical artificial intelligence. Inf. Fusion **79**(3), 263–278 (2022). 10.1016/j.inffus.2021.10.007

[82] Pfeifer, B., Saranti, A., Holzinger, A.: Gnn-subnet: disease subnetwork detection with explainable graph neural networks. Bioinformatics **38**(S–2), 120–126 (2022). 10.1093/bioinformatics/btac478 10.1093/bioinformatics/btac478 36124793

[83] Holzinger, A.: The next frontier: AI we can really trust. In: Joint European Conference on Machine Learning and Knowledge Discovery in Databases, pp. 427–440 (2021). 10.1007/978-3-030-93736-2_33

[84] Carrington, A.M., Manuel, D.G., Fieguth, P.W., Ramsay, T., Osmani, V., Wernly, B., Benett, C., Hawken, S., McInnes, M., Magwood, O., Sheikh, Y., Holzinger, A.: Deep ROC analysis and auc as balanced average accuracy, for improved classifier selection, audit and explanation. IEEE Trans. Pattern Anal. Mach. Intell. **45**(1), 329–341 (2023). 10.1109/TPAMI.2022.3145392 35077357 10.1109/TPAMI.2022.3145392

[85] Angerschmid, A., Zhou, J., Theuermann, K., Chen, F., Holzinger, A.: Fairness and explanation in ai-informed decision making. Mach. Learn. Knowl. Extraction **4**(2), 556–579 (2022). 10.3390/make4020026

